# Long‐Term Safety and Efficacy of Repeat Treatments with DaxibotulinumtoxinA in Cervical Dystonia: Results from the ASPEN‐Open‐Label Study

**DOI:** 10.1002/mdc3.70104

**Published:** 2025-05-29

**Authors:** Peter McAllister, Atul T. Patel, Marta Banach, Aaron Ellenbogen, Jaroslaw Slawek, Sebastian Paus, Hyder A. Jinnah, Virgilio Evidente, Todd M. Gross, Rashid Kazerooni, Conor J. Gallagher, David A. Hollander

**Affiliations:** ^1^ New England Institute for Neurology and Headache Stamford Connecticut USA; ^2^ Kansas City Bone & Joint Clinic Overland Park Kansas USA; ^3^ Jagiellonian University Krakow Poland; ^4^ Quest Research Institute Farmington Hills Michigan USA; ^5^ Department of Neurological‐Psychiatric Nursing, Faculty of Health Sciences Medical University of Gdansk Gdańsk Poland; ^6^ Neurology and Stroke Department St. Adalbert Hospital Gdańsk Poland; ^7^ Department of Neurology University of Bonn Bonn Germany; ^8^ Department of Neurology GFO Kliniken Troisdorf Germany; ^9^ Emory University School of Medicine Atlanta Georgia USA; ^10^ Movement Disorders Center of Arizona Scottsdale Arizona USA; ^11^ Revance Therapeutics, Inc. Nashville Tennessee USA

**Keywords:** cervical dystonia, movement disorders, spasmodic torticollis, botulinum toxins type A, daxibotulinumtoxinA

## Abstract

**Background:**

DaxibotulinumtoxinA (DAXI), a novel botulinum neurotoxin (BoNT) formulation, was shown to be safe, effective, and long‐lasting in the treatment of cervical dystonia (CD) over one treatment cycle in the phase 3, randomized, placebo‐controlled ASPEN‐1 trial.

**Objectives:**

To evaluate the safety, immunogenicity, and efficacy of repeat DAXI treatments for CD over 52 weeks in the phase 3, open‐label ASPEN‐OLS (NCT03617367).

**Methods:**

Adults with moderate‐to‐severe CD (Toronto Western Spasmodic Torticollis Rating Scale [TWSTRS] score ≥20) initially received DAXI 125U or 250U based on treatment history and investigator judgment. Retreatment could be titrated (50U‐75U) each cycle (maximum 300U) for up to four cycles over 52 weeks. Assessments were conducted at Week 4, 6, 12, and every 4 weeks until retreatment.

**Results:**

In all, 357 subjects received ≥1 dose of DAXI; most subjects (68.9%) received 250U during Cycle 1. Subjects most commonly received three (47.3%) or two (26.6%) treatments over 52 weeks. The average dose increased with successive cycles (Cycle 2: 239U, Cycle 3: 256U, Cycle 4: 270U). Mean (SD) change in TWSTRS score from baseline increased from −15.4 (10.3) in Cycle 1 to −19.9 (13.6) in Cycle 4. Median duration of effect was 20.1 weeks (Cycle 1, 2). No trend was observed between exposure to DAXI and any safety signals or antibody events. The most frequently reported treatment‐related adverse events per treatment were muscular weakness (4.9%), injection‐site pain (4.2%), and dysphagia (3.9%).

**Conclusion:**

DAXI was safe and efficacious over repeated treatments in adults with CD. Adverse event rates were similar to or potentially lower compared with conventional BoNTs.

Cervical dystonia (CD) is characterized by abnormal head and neck posture and painful spasms of the head and neck that require long‐term intervention and regular treatments.^1^, ^2^, ^3^ Repeat injection of botulinum toxin (BoNT) is first‐line treatment for CD,^2^ but there remains a significant unmet need based on limitations with conventional BoNT therapy.^4^, ^5^ Studies with the currently available conventional BoNT therapy have consistently demonstrated that patients begin to experience symptoms at an average of 10 weeks or sooner, while label restrictions, reimbursement policies, and historical concerns associated with neurotoxin immunogenicity have limited the minimum retreatment interval to 12 weeks,^6^, ^7^, ^8^, ^9^, ^10^ although some providers have pursued injections earlier than 12 weeks via prior authorizations, letters of medical necessity, and other documentation practices. As a result, a majority of patients with CD become symptomatic before their next treatment,^4^, ^10^ negatively impacting patient satisfaction, quality of life, and activities of daily living.^3^, ^4^ It has been well documented that patients with CD report higher levels of satisfaction with symptom control at peak toxin effect than at the end of their treatment cycle.^5^, ^6^, ^10^ Ultimately, patients with longer durations of effect have higher levels of satisfaction with their treatment,^5^ minimizing the symptomatic “rollercoaster” pattern many patients experience throughout the treatment cycle.^4^

Conventional BoNT products, which are formulated using the excipient human serum albumin (HSA) in addition to a salt or sugar, tend to have similar clinical profiles.^6^, ^11^, ^12^ DaxibotulinumtoxinA for Injection (DAXI; DAXXIFY®, Revance Therapeutics, Inc., Nashville, TN) is a novel formulation of BoNT type A without HSA, which contains a custom‐engineered peptide (RTP004) and was US Food and Drug Administration (FDA)‐approved in August 2023 for the treatment of CD in adults.^13^ RTP004 has been shown to augment the activity of the DAXI core neurotoxin by facilitating greater neuronal cell binding, increasing neuronal bioavailability and SNAP‐25 cleavage, and anchoring the neurotoxin to the target tissue.^14^, ^15^, ^16^ In addition, RTP004 helps to prevent surface adsorption and increases the thermostability of the formulation.^17^

In the phase 3 ASPEN‐1 clinical trial, both the 125U and 250U doses of DAXI were shown to be safe and effective in the treatment of CD.^18^ In addition to a long duration of effect (median, 20–24 weeks), the ASPEN‐1 study demonstrated lower rates of dysphagia and muscle weakness (both <5%) compared with previously reported CD BoNT trials.^6^, ^11^, ^12^, ^19^, ^20^ Due to the chronic nature of CD, additional information on dose optimization and on the safety, immunogenicity, and efficacy of repeat doses of DAXI across successive treatment cycles is needed. The ASPEN‐OLS trial aimed to assess the long‐term safety, immunogenicity, and efficacy of repeat DAXI treatments across 52 weeks.

## Methods

### Study Design

**Figure 1:**
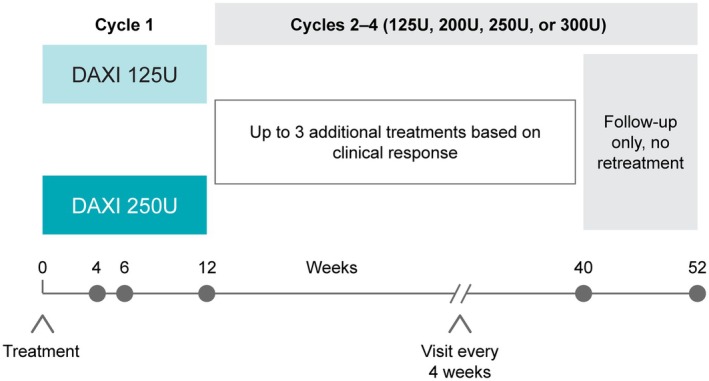
Study design. DAXI, daxibotulinumtoxinA.

ASPEN‐OLS was a phase 3, open‐label extension trial conducted at 64 sites in nine countries (Austria, Canada, Czech Republic, France, Germany, Poland, Spain, United Kingdom, and United States) from August 2018 to May 2021 (Fig. 1). Ethics approval was obtained by all study sites, and the study was conducted according to the principles of the Declaration of Helsinki and applicable laws and regulations. All subjects provided written informed consent. The study is registered at www.clinicaltrials.gov (NCT03617367).

### Study Population

Adults aged 18 to 80 years who met diagnostic criteria for isolated CD, with a Toronto Western Spasmodic Torticollis Rating Scale (TWSTRS) total score ≥20 (subscale scores: severity ≥15, disability ≥3, and pain ≥1) were eligible. Eligible patients included those who completed the prior ASPEN‐1 trial,^18^ were naïve to BoNT treatment, or were BoNT‐treatment experienced patients with a demonstrated response to their last BoNT treatment. The main exclusion criteria were CD that was attributable to an underlying etiology or the presence of predominant retrocollis or anterocollis.

### Treatment Protocol

After screening, subjects received their initial intramuscular injections of DAXI (Day 1 of Cycle 1). The initial dose was 250U for subjects who had previously received ≥190U of onabotulinumtoxinA or incobotulinumtoxinA equivalent, or for those who would qualify for a dose of ≥190U based on investigator judgment. Patients on abobotulinumtoxinA could also be enrolled and were dosed based on investigator judgment. All others received an initial dose of 125U. All ASPEN‐OLS treatments were open‐label. The randomly assigned treatment from ASPEN‐1 remained masked for subjects from that earlier study.

Reconstituted DAXI (2.5 mL in volume) was divided for injection into involved muscles (levator scapulae, longissimus capitis and cervicis, scalenus complex, splenius capitis, splenius cervicis, sternocleidomastoid, and trapezius, as needed), as determined by the investigator based on each subject's clinical presentation. Use of electromyography, ultrasound, or other modalities to guide injection was optional. After DAXI was administered on Day 1, participants were followed up by telephone after 2 weeks, and by clinical visits after 4, 6, and 12 weeks, and every 4 weeks thereafter until retreatment.

Retreatment cycles were limited to a minimum of 12 weeks (±14‐day treatment window), with a maximum of four cycles of DAXI that could be administered within the 52‐week study. Subjects were eligible for retreatment after they had reached the minimum residual benefit (MRB),^18^ also known as the target TWSTRS,^20^, ^21^ defined as the loss of 80% of improvement in TWSTRS total score averaged for Week 4 and 6. Subjects could also request retreatment, with investigator agreement, prior to reaching the MRB if they experienced clinically meaningful recurrence of CD symptoms. Retreatment was not allowed after Week 40 to allow for a minimum follow‐up period of 12 weeks in the final treatment cycle.

Beginning with Cycle 2, the administered dosage could be titrated based on the subject's response. The four pre‐defined dose levels were DAXI 125U, 200U, 250U, and 300U. The doses were increased or decreased by a maximum of one pre‐defined dose step (50U–75U) for each cycle.

### Outcome Measures and Endpoints

The primary objectives were to evaluate the safety and immunogenicity of long‐term DAXI treatment. Safety endpoints included the incidence of treatment‐related treatment‐emergent adverse events (TEAEs), discontinuations due to treatment‐related TEAEs, and treatment‐emergent immunogenicity by treatment cycle and dose. Assessment of immunogenicity included serum antibody tests for BoNT type A and for RTP004. Blood samples for antibody testing were collected at screening prior to any retreatment, Week 4 of each treatment cycle, and the Week 52 or end of study visit. Blood samples were tested for the presence of binding antibodies to DAXI or the RTP004 peptide by separate enzyme‐linked immunosorbent assay methods (ELISA). Samples found to be positive for the presence of binding antibodies to DAXI were evaluated for neutralizing antibodies in the mouse protection assay.

The secondary endpoints included evaluation of the long‐term efficacy of successive DAXI treatments and the duration of effect. These endpoints included change from baseline in the TWSTRS total score averaged at Week 4 and 6 within each cycle. The TWSTRS scale ranges from 0 to 85 and comprises three subscales: severity (0–35), disability (0–30), and pain (0–20), with higher scores indicating greater impairment.^22^ The magnitude of change was evaluated for pooled dosages in each treatment cycle.

The percentage of responders in Patient Global Impression of Change (PGIC) and Clinical Global Impression of Change (CGIC) was also assessed at Week 4 and 6 of each treatment cycle. The PGIC and CGIC evaluate the global response to treatment on a 7‐point scale from very much worse (−3) to very much better (+3). Those who responded moderately better (+2) or very much better (+3) were considered responders.

Duration of effect was defined as the time from treatment until loss of ≥80% of peak treatment effect, consistent with prior studies,^20^, ^21^ and was evaluated for treatment Cycles 1 and 2, which were not artificially truncated in this 52‐week study. A second, related endpoint, time to retreatment, was defined as the time to receive a retreatment, whether by request or by loss of 80% of effect.

### Statistical Analysis

The study was designed to collect safety data for at least 350 DAXI‐treated subjects. The sample size was chosen to ensure approximately 100 subjects would receive three or four continuous doses based on anticipated retreatment intervals and potential for dropouts from the study.

All safety and efficacy analyses were performed on the safety population, defined as enrolled subjects who received at least one dose of study drug. Efficacy results were summarized with descriptive statistics. For CGIC and PGIC responders, missing data were set to 0 (ie, about the same) and there was no imputation for those who completed or discontinued the study. Analyses were conducted using SAS version 9.4 (SAS Institute, Cary, NC). Kaplan–Meier analyses were performed to produce survival curves and median time to event to evaluate duration of effect (ie, time to loss of 80% of effect) and time to retreatment or loss of 80% effect, for Cycles 1 and 2. For time to loss of 80% of effect, subjects who completed, discontinued, or were retreated prior to reaching their target TWSTRS score were censored (ie, included until the retreatment date or end of study date). Those with no improvement at Week 4 were assigned a duration of 0. Time to retreatment or loss of 80% of effect was analyzed using the same method, except that subjects who were retreated prior to loss of 80% were counted as having reached the target event. Post hoc analyses of the use of guided injections were performed for peak efficacy using an independent t‐test with site as the unit of analysis, for duration of effect and time to retreatment using a Kaplan–Meier log‐rank test, and for incidence of adverse events using Fisher's exact test.

## Results

### Study Population, Treatment Exposure, and Dosing

**Figure 2:**
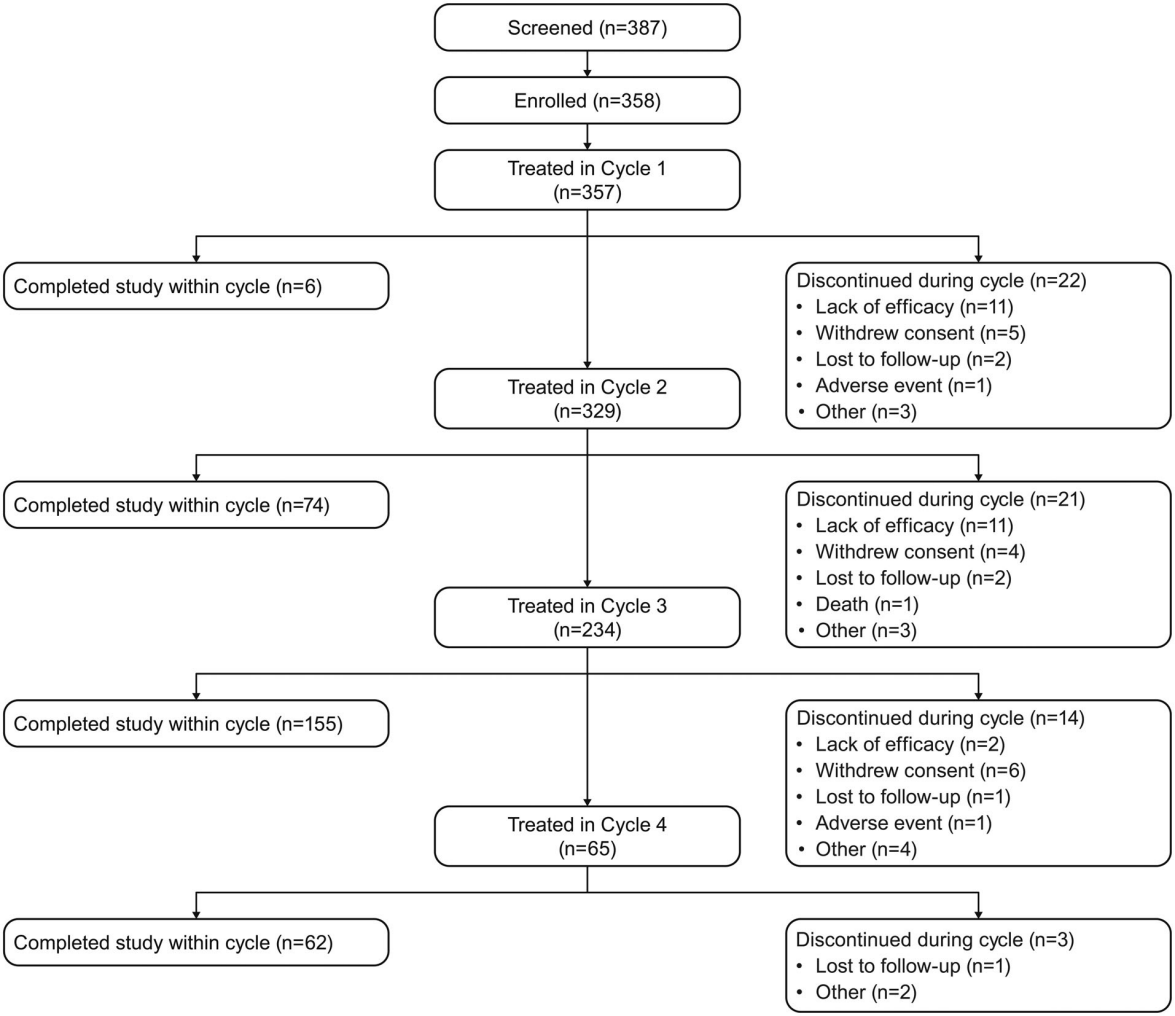
Subject flow.

Of 387 subjects with CD who were screened, 358 (including 86 new participants who did not participate in the ASPEN‐1 trial) were enrolled, and the 357 who received at least one dose of DAXI were included in the analysis (Fig. 2). Most (83.2%) subjects reached the end of the study at 52 weeks. The two most common reasons for early discontinuation were lack of efficacy (6.7% of those enrolled) and withdrawal of consent (4.2%) (Fig. 2). Most subjects were female (66.7%), White (95.8%), and had received prior treatment with BoNT (81.5%) (Table 1). Overall, 985 doses of DAXI were administered over the 52‐week study; 28 (7.8%) subjects received one treatment, 95 (26.6%) received two treatments, 169 (47.3%) received three treatments, and 65 (18.2%) received four treatments. A total of 582 (59.1%) of 985 treatments at 43 of 64 investigational sites used guidance for the injections (electromyography, ultrasound, or other); the specific type of guidance was not collected.

**TABLE 1:** Demographics and baseline characteristics (safety population)

Variable	Treatment cycle 1 dose group		All subjects (*N* = 357)
	DAXI 125 U (*N* = 111)	DAXI 250U (*N* = 246)	
Age, years			
Mean (SD)	56.5 (13.0)	58.1 (11.3)	57.6 (11.9)
[min, max]	[25, 80]	[19, 80]	[19, 80]
Female, *n* (%)	90 (81.1)	148 (60.2)	238 (66.7)
Race, *n* (%)			
White	106 (95.5)	236 (95.9)	342 (95.8)
Black	1 (0.9)	5 (2.0)	6 (1.7)
Asian	2 (1.8)	2 (0.8)	4 (1.1)
Other^a^	2 (1.8)	3 (1.2)	5 (1.4)
Ethnicity, *n* (%)			
Hispanic or Latino	6 (5.4)	13 (5.3)	19 (5.3)
Not provided	2 (1.8)	3 (1.2)	5 (1.4)
Prior treatment with BoNT, *n* (%)	67 (60.4)	224 (91.1)	291 (81.5)
Baseline TWSTRS score, mean (SD)	41.2 (9.9)	44.3 (10.1)	43.3 (10.1)
Baseline BMI, mean (SD), kg/m^2^	26.4 (5.3)	27.8 (5.4)	27.4 (5.4)

Abbreviations: BMI, body mass index; BoNT, botulinum neurotoxin; DAXI, daxibotulinumtoxinA; max, maximum; min, minimum; *n*, number of participants in category; SD, standard deviation; TWSTRS, Toronto Western Spasmodic Torticollis Rating Scale.

^a^ Other includes American Indian or Alaska Native (one in the DAXI 250U group), Native Hawaiian or Other Pacific Islander (one in the DAXI 250U group) and other (two in the 125U group and one in the 250U group).

**Figure 3:**
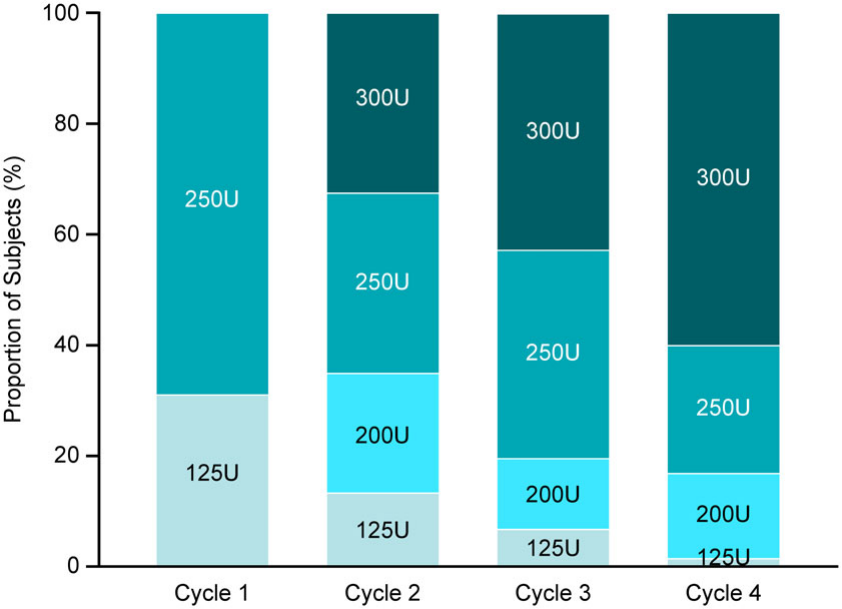
Summary of DAXI starting dose and dosing administration across Cycles 1–4. DAXI, daxibotulinumtoxinA.

Most subjects (68.9%) received DAXI 250U at Cycle 1 (Fig. 3). Of the 66 subjects who were BoNT‐naive, 22 (33.3%) started on 250U and 44 (66.7%) started on 125U. Overall, the mean DAXI dose increased with each successive cycle from 211U in Cycle 1 to 239U in Cycle 2, 256U in Cycle 3, and 270U in Cycle 4. Of the 65 (18.2%) subjects who received the maximum total of four cycles, most (60.0%) received 300U by Cycle 4 (Fig. 3).

### Safety

A summary of the TEAEs based on treatment cycle is listed in Table 2. During the study, 122 (34.2%) subjects reported 278 treatment‐related TEAEs. The frequency of treatment‐related TEAEs in Cycle 1 was 21% and remained low in successive treatment cycles (Table 2). The most commonly reported treatment‐related TEAEs (occurring in ≥3% of subjects) were dysphagia (4.2% of treatments), muscular weakness (4.9% of treatments), injection‐site pain (2.7% per treatment), injection‐site erythema (2.2% per treatment), and neck pain (1.2% per treatment). All events of muscular weakness were localized to the injection areas. The rates of dysphagia and muscle weakness remained low at all dosing intervals from 12 weeks to 25 weeks or more. Among the 16 instances in which retreatments were performed at 11 weeks following an earlier treatment, there were two cases of dysphagia and three cases of muscle weakness involving a total of four subjects. Serious TEAEs were reported by 17 (4.8%) subjects, with a similar incidence across doses; none were considered related to study treatment. One subject died during the study with a serious TEAE of chronic kidney disease, which was considered not related to study treatment. Three subjects (0.8%) discontinued the study because of a TEAE (*n* = 1 each); of these, two TEAEs were considered related to study treatment (injection‐site erythema and hematoma). A post hoc analysis did not identify any statistical differences in the incidence of key TEAEs, such as dysphagia or muscle weakness, between injections with guidance and injections without guidance (*P* > 0.05).

**TABLE 2:** Summary of TEAEs by DAXI treatment cycle

Variable	Cycle 1 (*N* = 357)	Cycle 2 (*N* = 329)	Cycle 3 (*N* = 234)	Cycle 4 (*N* = 65)	Overall (*N* = 357)	Rate per treatment (%)^a^
Subjects with TEAEs, *n* (%)						
Any TEAE	172 (48.2)	134 (40.7)	98 (41.9)	22 (33.8)	245 (68.6)	–
Serious TEAEs	2 (0.6)	6 (1.8)	8 (3.4)	1 (1.5)	17 (4.8)	–
TEAEs leading to study discontinuation	1 (0.3)	0	2 (0.9)	0	3 (0.8)	–
TEAEs leading to death	0	1 (0.3)	0	0	1 (0.3)	–
Subjects with treatment‐related TEAEs, *n* (%)						
Any treatment‐related TEAE^b^	75 (21.0)	56 (17.0)	46 (19.7)	9 (13.8)	122 (34.2)	–
Dysphagia	14 (3.9)	14 (4.3)	11 (4.7)	2 (3.1)	35 (9.8)	4.2
Muscular weakness	15 (4.2)	15 (4.6)	15 (6.4)	2 (3.1)	31 (8.7)	4.9
Injection site pain	15 (4.2)	7 (2.1)	2 (0.9)	2 (3.1)	22 (6.2)	2.7
Injection site erythema	8 (2.2)	6 (1.8)	7 (3.0)	1 (1.5)	15 (4.2)	2.2
Neck pain	5 (1.4)	4 (1.2)	3 (1.3)	0	11 (3.1)	1.2

*Note*: Treatment‐related musculoskeletal pain: Cycle 1, two subjects and two events; Cycle 2, 0 events; Cycles 3 and 4, one subject and one event.

Abbreviations: DAXI, daxibotulinumtoxinA; TEAE, treatment‐emergent adverse event.

^a^ 985 treatments.

^b^ Occurring in ≥3% of subjects and *n* ≥ 3 subjects in any group.

### Immunogenicity

Of subjects with evaluable samples at baseline (*n* = 343), only two (0.6%) developed binding antibodies to BoNT type A across all treatment cycles for the study duration. One of these two subjects had developed neutralizing antibodies, assessed by the mouse protection assay, to BoNTA in the ASPEN‐1 study and remained positive for neutralizing antibodies in ASPEN‐OLS, despite exhibiting a clinical response in this study. This subject was diagnosed with CD in 1994 and had been treated with rimabotulinumtoxinB since 1996. Binding antibodies to the RTP004 peptide developed in 1.2% (4/343) of subjects, and all of these subjects exhibited a clinical response to DAXI.

### Efficacy

**Figure 4:**
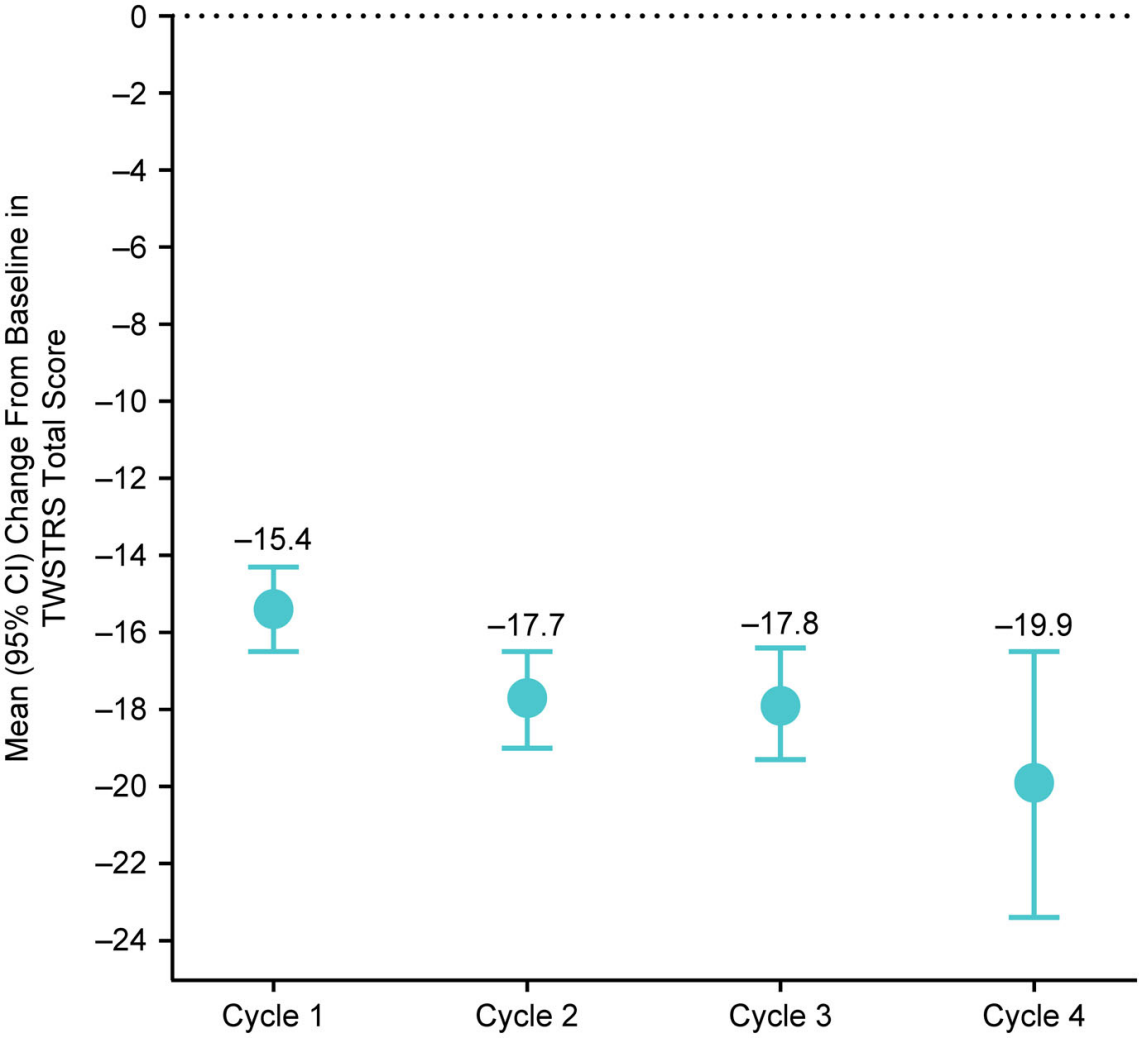
Mean change from baseline in TWSTRS total score averaged at Weeks 4 and 6 for each treatment cycle. CI, confidence interval; TWSTRS, Toronto Western Spasmodic Torticollis Rating Scale.

The mean change from baseline in TWSTRS total score averaged for Week 4 and 6 improved from Cycle 1 to Cycle 4 as the dose was titrated (Fig. 4). The mean (SD) improvement in TWSTRS total score from baseline at Week 4 and 6 was −15.4 (10.3) in Cycle 1, −17.7 (11.4) in Cycle 2, −17.9 (11.3) in Cycle 3, and −19.9 (13.6) in Cycle 4. Mean reductions in TWSTRS pain, disability, and severity subscale scores followed a similar pattern, and continued to improve from Cycle 1 to Cycle 4. CGIC response rates increased from 72.3% in Cycle 1 to 81.8% in Cycle 2 and were maintained through Cycle 3 (78.2%) and 4 (83.1%). The PGIC response rates were 67.2% in Cycle 1 and remained relatively constant through Cycle 2 (71.7%), 3 (65.0%), and 4 (69.2%). A post hoc analysis indicated that injections with guidance did not result in greater peak efficacy than injections without guidance (*P* > 0.05).

### Duration of Effect and Time to Retreatment

**Figure 5:**
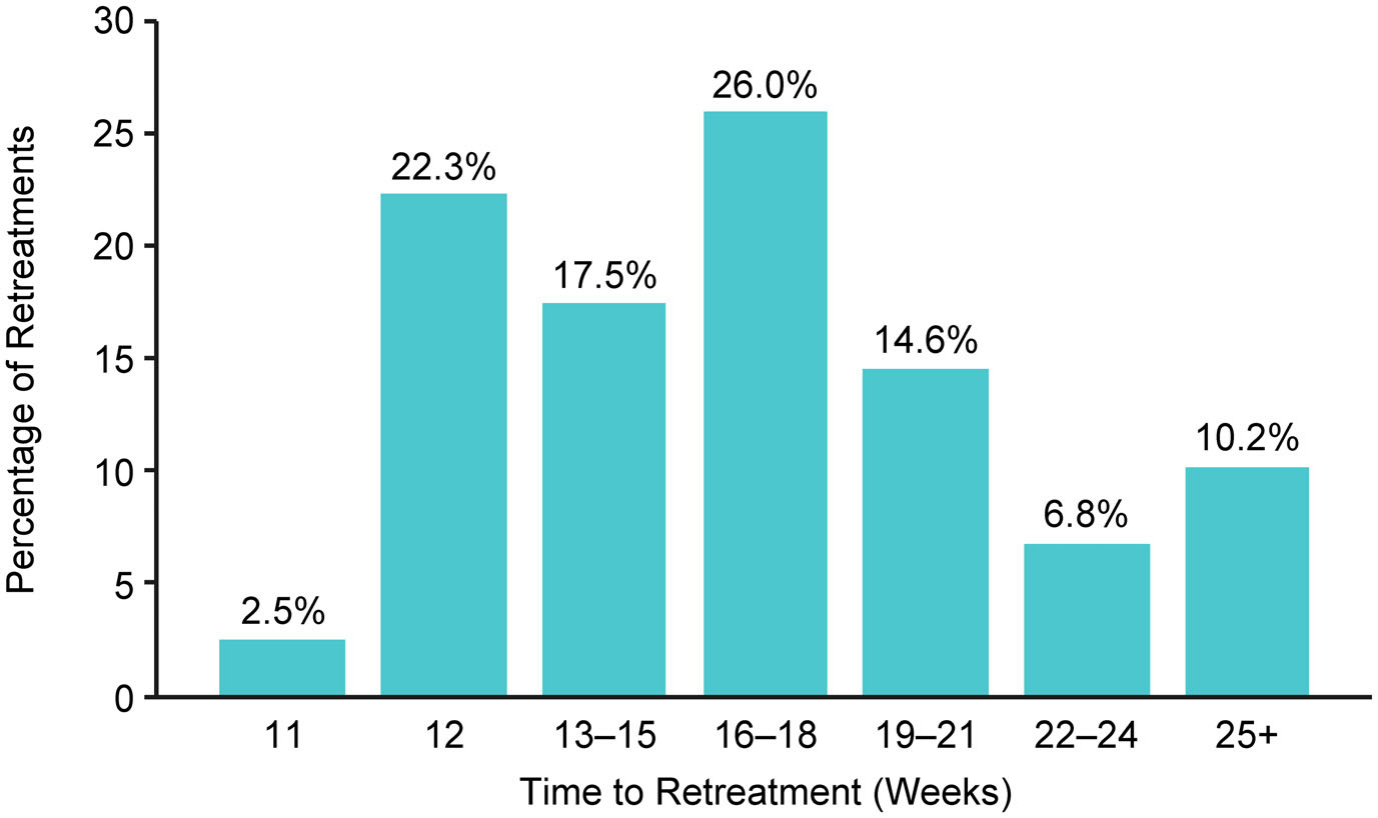
Histogram of all times to retreatment with DAXI (628 treatments). DAXI, daxibotulinumtoxinA.

Based on time to loss of 80% of peak effect, the median duration of effect observed was 20.1 weeks (95% CI 19.3, 21.7) in Cycle 1 (*n* = 357) and 20.1 weeks (95% CI 19.7, 24.0) in Cycle 2 (*n* = 329). As permitted by study protocol, 264 (28.7% of total treatments) subject requests for retreatment occurred before 80% loss of effect was reached following either Cycle 1, 2, or 3. The median time to retreatment based on request or time to loss of 80% of effect was 17.1 (95% CI 16.6, 19.1) weeks in Cycle 1 (*n* = 357) and 16.9 (95% CI 16.1, 17.3) weeks in Cycle 2 (*n* = 329). A post hoc analysis indicated no difference in duration of effect or time to retreatment (both *P* > 0.05) for injections performed with versus without guidance. A summary of the intervals between all retreatments (*n* = 628) administered during this study is provided in Figure 5. The majority of subjects (57.6%) received retreatments at 16 weeks or later, as doses were titrated.

## Discussion

This 52‐week study demonstrates the sustained efficacy and safety of DAXI across repeat treatments for CD. DAXI is formulated with a custom‐engineered peptide (RTP004) that facilitates increased neuronal membrane binding and greater toxin bioavailability.^14^, ^15^, ^16^ As such, DAXI was introduced as a longer‐lasting neurotoxin to provide an alternative option for patients with CD, in particular those patients who experience early symptom re‐emergence while being treated with conventional BoNTs with standard 12‐week dosing intervals.

During this study, physicians were able to titrate the total dosage of DAXI as needed based on fixed predetermined increments. Approximately 70% (246/357) of patients were started on 250U, based on either clinical severity of CD or protocol guidance of prior treatment with ≥190U of onabotulinumtoxinA or incobotulinumtoxinA equivalent. For patients who were BoNT‐naïve, one‐third were started on 250U and two‐thirds were started on 125U. Overall, the mean total dose increased over the course of the treatment cycles, from a mean of 211U at Cycle 1 to a mean of 270U by Cycle 4. As the dose was optimized, the magnitude of improvement in TWSTRS total score continued to increase from −15.4 in Cycle 1 to −19.9 in Cycle 4. These improvements were also greater than those observed (−12.6) after a single treatment in the same patients who had also been randomized to either 125U or 250U in ASPEN‐1. Titration across several cycles, as seen in this study with DAXI, has also been well documented with other BoNTs, as physicians steadily optimize efficacy while watching for any safety signals.^23^, ^24^

This is the first study to investigate repeat treatments of DAXI for treatment of CD. The low rates of treatment‐related TEAEs seen with DAXI in this study, even as doses were titrated, were consistent with those reported in ASPEN‐1.^18^ In addition, the rates of dysphagia and muscular weakness in this study were favorable relative to the rates of dysphagia and muscular weakness previously reported in pivotal trials of other FDA‐approved BoNTs for CD (dysphagia: 13–39%, muscle weakness: 7–15% for approved doses up to 500U).^6^, ^11^, ^12^, ^19^, ^20^, ^25^ A relatively low rate of injection‐site pain (2.7% per treatment) was also observed. It is possible that some patients may have experienced transient injection pain related to the presence of a weak buffer in the formulation. This buffer is important in the extended (72‐h) post‐reconstitution stability of DAXI.^17^

The low rates of adverse events, even as doses were titrated higher throughout the course of the 52‐week study, may be a direct result of the DAXI formulation, which is thought to minimize unwanted spread to off‐target tissues.^14^, ^15^, ^16^ The novel, custom‐engineered peptide (RTP004) in the DAXI formulation is highly positively charged, which drives electrostatic binding to negatively charged extracellular structures, such as neuronal surfaces and extracellular matrix proteins.^16^ This enhanced binding may facilitate the localization of DAXI neurotoxin and reduce diffusion from the injection site, as was demonstrated in an animal study comparing DAXI to onabotulinumtoxinA.^26^ As a result of the enhanced neuronal binding, the amount of core neurotoxin necessary to be delivered with each treatment to achieve a desired effect may be less than that required with conventional BoNTs. For example, a 250U dose of DAXI contains 1.12 ng of core neurotoxin, whereas the labeled dose of 236U with onabotulinumtoxinA for CD contains 2.12 ng of core neurotoxin.^6^, ^27^ The lower levels of core neurotoxin needed with the DAXI formulation may also account for the low rates of immunogenicity observed, as only a single subject with a complex 25‐year history of BoNT use developed neutralizing antibodies during this study.

The median duration of effect for DAXI, measured as time until loss of 80% of peak effect based on TWSTRS total score, was approximately 20 weeks for both Cycle 1 and 2, consistent with ASPEN‐1,^18^ and was longer than the duration for onabotulinumtoxinA previously reported using a similar methodology.^20^ Many patients, however, prefer to be retreated when their symptoms begin to re‐emerge prior to returning to baseline.^10^ In this study, subjects’ requests for retreatment prior to their symptoms returning to baseline (based on 80% loss of efficacy) were observed after 28.7% of total treatments, highlighting the need to reduce the “rollercoaster” of symptoms many patients currently experience. Overall, subjects in the study were retreated between 11 and 41 weeks following a DAXI treatment, at a median of 17 weeks following each of the first two treatment cycles. During the entire 52‐week study, as doses were titrated, the majority (57.6%) of subjects received retreatment at 16 weeks or later. This extended dosing interval is especially noteworthy given the lower amount of active toxin administered per average DAXI treatment relative to conventional BoNTs, and further illustrates the beneficial effects of the formulation in facilitating increased neuronal membrane binding, neuronal bioavailability, and ultimately greater SNAP‐25 cleavage.^14^, ^15^, ^16^

The findings of this study serve as a potential template for real‐world dose optimization, as physicians selected doses based on clinical presentation and prior BoNT history and titrated the doses based on clinical response. Surveys have demonstrated that up to two‐thirds of patients do not achieve 3 months of adequate symptom relief with standard intervals on the currently available BoNTs.^4^ Long‐acting DAXI may be especially well suited to address the re‐emergence of symptoms in these patients. The need for better symptom control over the course of the entire treatment interval has been highlighted in another flexible‐interval open‐label study of incobotulinumtoxinA in which almost half of patients requested injection intervals less than every 12 weeks, with 22.5% requesting reinjection within 6–10 weeks.^25^ In the current study, most subjects delayed their dosing intervals with DAXI beyond the standard 12 weeks, despite the fact that the DAXI dose was not yet optimized over the 52 weeks. The long duration of DAXI may allow physicians to provide their patients with more sustained symptom control over a similar 12‐week reinjection interval compared with their current BoNT treatment or further extend the treatment intervals through dose optimization based on patient needs.

This open‐label safety study supports the findings seen in the ASPEN‐1 trial,^18^ though several limitations should be noted. Consistent with the protocol design, the number of cycles of treatment each subject received varied. As reinjection was based on decline of treatment effectiveness, subjects with longer durations of effect typically did not reach Cycle 4. The relatively low proportion of patients treated in Cycle 4 (*n* = 65; 18.2% of enrolled patients) contributed to the larger 95% CI in this cycle compared with previous cycles. In addition, the doses were limited in this study to specific dose levels ranging from 125U to 300U. Doses greater than 300U may be required for adequate symptom control for many patients, especially those currently on high doses of conventional BoNTs. Of note, doses as high as 450U of DAXI in CD were well tolerated in previous studies.^28^ Additionally, initial dosing was fixed at 125U or 250U. Although this was based on patients’ previous BoNT dose, this two‐option fixed‐dose starting point will not reflect the wider range of initial starting doses to be used in real world clinical practice. Within this design, it was also not possible to demonstrate a specific dose‐related response for efficacy or duration. We also did not specifically assess non‐motor symptoms. Lastly, although patients with predominant anterocollis and retrocollis were excluded from this study, it should be noted that patients with these types of CD can respond well to BoNT treatment.^29^

This study demonstrated that DAXI was safe and efficacious with repeat treatments in adults with CD, with no increasing incidence of treatment‐related TEAEs across treatment cycles, even during dose titration. In addition, the DAXI formulation appears to offer an extended duration of effect and offers a new option for physicians to potentially provide patients with improved symptom relief with either a standard 12‐week or longer dosing interval.

## Author Roles

(1) Research project: A. Conception, B. Organization, C. Execution; (2) Statistical Analysis: A. Design, B. Execution, C. Review and Critique; (3) Manuscript Preparation: A. Writing of the first draft, B. Review and Critique.

P.M.A.: 1C, 3B.

A.T.P.: 1A, 1B, 1C, 2C, 3B.

M.B.: 1C 2C, 3B.

A.E.: 1C, 3B.

J.S.: 3B.

S.P.: 1C, 2C, 3B.

H.A.J.: 1C, 2C, 3B.

V.E.: 1C, 3B.

T.M.G.: 1A, 1B, 1C, 2A, 2B, 2C, 3B.

R.K.: 2C, 3A, 3B.

C.J.G.: 2C, 3A, 3B.

D.A.H.: 2C, 3A, 3B.

## Disclosures

**Ethical Compliance Statement:** The protocol for this study was approved by the appropriate national and local authorities and independent ethics committees or institutional review boards at each study site. Written informed consent was obtained from all participants who were enrolled in the study. We confirm that we have read the Journal's position on issues involved in ethical publication and affirm that this work is consistent with those guidelines.

**Funding Sources and Conflict of Interest:** Revance Therapeutics, Inc. funded the study and was involved in the study design, collection, analysis, and interpretation of the data; writing of the report; and decision to submit the report for publication. The authors did not receive honoraria or payments for authorship. TMG, RK, CJG, and DAH are employees or former employees of Revance Therapeutics.

**Financial Disclosures for the Previous 12 Months:** PMA has served as a consultant and/or on speaker bureaus for AbbVie, ANI, Axsome, Eli Lilly, Merz, Pfizer and Revance; and has received research funding from AbbVie, Axsome, Eli Lilly, Ipsen, Lundbeck, Merz, Neurocrine, Novartis, Pfizer, and Revance. ATP has received research grants from AbbVie, AEON, and Ipsen; and has served as a consultant and/or speaker for AbbVie, IPSEN, and Revance. MB has served as a consultant and advisory board member for Merz. AE has served as a consultant and/or speaker for AbbVie, Amneal, Ipsen, Medtronic, Neurocrine, Revance, Sunovion, and Teva; and has received research support from AbbVie, Acadia, AEON, Aptinyx, Cerevance, CND Life Sciences, Ipsen, IRLAB, Jazz Pharmaceuticals, Neurocrine, Revance, Scion Neurostim, and Theravance. JS has received honoraria for lectures and advisory board participation from AbbVie, Ipsen, and Merz. SP is employed by GFO Clinics Troisdorf; has served as a consultant and/or speaker for AbbVie, Bial, Bayer, Idorsia, Ipsen, Merz, Novartis, and Teva; and has been involved in clinical trials (Phase II and III) for Bayer, Ipsen, and Syneos Health. HAJ has grant support (recent, active, or pending) from the US government (National Institutes of Health), and from AbbVie, Addex, AEON, Ipsen, Revance, Sage, and Jazz Pharmaceuticals. He has served on advisory boards and/or as a consultant for Addex, AbbVie, Apello, Atlas, Ipsen, PureTech, Revance, Takaha/Ene, and Vima; and has received honoraria or stipends for lectures or administrative work from the International Parkinson's Disease and Movement Disorders Society. He has also served on scientific advisory boards for several private foundations including the Benign Essential Blepharospasm Research Foundation, Cure Dystonia Now, the Dystonia Medical Research Foundation, and the Tourette Association of America. HAJ has served as a principal investigator for the Dystonia Coalition, which has received the majority of its support through the NIH (grants NS116025, NS065701 from the National Institutes of Neurological Disorders and Stroke TR001456 from the Office of Rare Diseases Research at the National Center for Advancing Translational Sciences). The Dystonia Coalition has received additional material or administrative support from industry sponsors (Allergan Inc. and Merz Pharmaceuticals) as well as private foundations (The Benign Essential Blepharospasm Foundation, Cure Dystonia Now, The Dystonia Medical Research Foundation, The National Spasmodic Dysphonia Association, and the National Spasmodic Torticollis Association). VE has served as a consultant and/or speaker for AbbVie, Amneal, Ipsen, Medtronic, Neurocrine, Revance, Sunovion, and Teva; and has received research support from AbbVie, Acadia, AEON, Aptinyx, Cerevance, CND Life Sciences, Ipsen, IRLAB, Jazz Pharmaceuticals, Neurocrine, Revance, Scion Neurostim, and Theravance.

## Supporting information


**Supplemental File.** ASPEN Study Investigators.

## Data Availability

The data reported are part of a global, sponsor‐led clinical development and registration program. Anonymized data not provided in this manuscript may be shared at the request of any qualified investigator.
