# Supplementary material for: Long‐Term Safety and Efficacy of Repeat Treatments with DaxibotulinumtoxinA in Cervical Dystonia: Results from the ASPEN‐Open‐Label Study
Source: Mov Disord Clin Pract. 2025 May 29;12(11):1764–73. doi: 10.1002/mdc3.70104 (PMC12625142; doi:10.1002/mdc3.70104)
Supplement: Supplementary file 1 — Supplemental File. ASPEN Study Investigators. [file MDC3-12-1764-s001.docx]

**Supplemental File**. ASPEN Study Investigators.

| **Name** | **Location** | |
| --- | --- | --- |
| Philipp Albrecht | Düsseldorf, Germany | |
| Richard Barbano | Rochester, NY, USA | |
| Norman Bettle | Port Royal, SC, USA | |
| Sylvia Bösch | Innsbruck, Austria | |
| Francoise Bouhour | Bron, France | |
| James Boyd | Burlington, VT, USA | |
| Giovanni Castelnovo | Nimes, France | |
| Pratap Chand | St Louis, MO, USA | |
| Susan Criswell | St. Louis, MO, USA | |
| Khashayar Dashtipour | Loma Linda, CA, USA | |
| Andres Deik | Philadelphia, PA, USA | |
| Aaron Ellenbogen | Farmington, MI, USA | |
| Virgilio Evidente | Scottsdale, AZ, USA | |
| Danielle Feigenbaum | Los Angeles, CA, USA | |
| Susan Fox | Toronto, ON, Canada | |
| JeffreyzGitt | Phoenix, AZ, USA | |
| John Goudreau | East Lansing, MI, USA | |
| Timothy Harrower | Exeter, UK | |
| Bernhard Haslinger | Munich, Germany | |
| Daragh Heitzman | Dallas, TX, USA | |
| Jorge Hernandez-Vara | Barcelona, Spain | |
| David Isaacs | Nashville, TN, USA | |
| Stuart Isaacson | Boca Raton, FL, USA | |
| Robert Jech | Prague, Czech Republic | |
| Hyder Jinnah | Atlanta, GA, USA | |
| Christopher Kobylecki | Salford, UK | |
| Katja Kollewe | Hannover, Germany | |
| Aikaterini Kompoliti | Chicago, IL, USA | |
| Dariusz Koziorowski | Warszaw, Poland | |
| Alexandre Kreisler | Lille, France | |
| Rajeev Kumar | Englewood, NJ, USA | |
| Mark LeDoux | Memphis, TN, USA | |
| Peter LeWitt | West Bloomfield, MI, USA | |
| Ebba Lohmann | Tübingen, Germany | |
| Lydia Lopez Manzanares | Madrid, Spain | |
| Irene Malaty | Gainesville, FL, USA | |
| Janice Massey | Durham, NC, USA | |
| Peter McAllister | Stamford, CT, USA | |
| Elena Moro | Grenoble, France | |
| William Ondo | Houston, TX, USA | |
| Javier Pagonabarraga Mora | Barcelona, Spain | |
| Sebastian Paus | Troisdorf, Germany | |
| Elizabeth Peckham | Round Rock, TX, USA | |
| Rekha Pillai | Cordova, TN, USA | |
| Monika Rudzinska | | Krakow, Poland |
| Harvey Schwartz | | Hollywood, CA, USA |
| Carlos Singer | | Miami, FL, USA |
| Jaroslaw Slawek | | Gdansk, Poland |
| Dariusz Szabela | | Warsaw, Poland |
| Jessica Tate | | Winston-Salem, NC, USA |
| Diego Torres | | Omaha, NE, USA |
| Jose Maria Trejo | | Burgos, Spain |
| Daniel Truong | | Fountain Valley, CA, USA |
| Winona Tse | | New York, NY, USA |
| Andrzej Tutaj | | Olsztyn, Poland |
| Alberto Vasquez | | St Petersburg, FL, USA |
| Oldrich Vysata | | Rychnov nad Kneznou, Czech Republic |
| Ivana Woznicova | | Ostrava-Poruba, Czech Republic |
| Sharon Yegiaian | | Pasadena, CA, USA |
